# The use of direct and indirect speech across psychological distance

**DOI:** 10.3758/s13421-021-01267-x

**Published:** 2022-01-14

**Authors:** Jianan Li, Katinka Dijkstra, Rolf A. Zwaan

**Affiliations:** Department of Psychology, Education and Child Studies, Erasmus University Rotterdam, Burgemeester Oudlaan 50, Mandeville Building, PO Box 1738, 3000DR Rotterdam, The Netherlands

**Keywords:** Direct speech, Indirect speech, Psychological distance, Construal level theory

## Abstract

The current study investigated how psychological distance affects people’s preference for direct and indirect speech in a narrative task. In three experiments, participants were instructed to first watch a video and then retell what happened in the video to an imagined/anticipated listener. We manipulated social distance (Experiment 1), temporal distance (Experiment 2), and spatial distance (Experiment 3) between participants and the listener. We compared the proportions of direct speech in the narrations from psychologically proximal versus distal conditions. Experiments 1 and 2 showed that social and temporal proximity increased the rates of direct speech. Social and temporal distance, conversely, increased the rates of indirect speech. Experiment 3 did not yield a significant difference in the use of direct and indirect speech between spatially proximal and distal conditions. Taken together, our results indicate that different psychological dimensions might have discrepant effects on people’s choices between direct and indirect speech. Possible explanations for the discrepancy among different psychological distance dimensions are discussed.

People communicate differently in different situations. Situational factors have been observed to affect both the content and linguistic-paralinguistic-nonverbal features of communication (Giles & Ogay, 2007). As regards content, situational factors play a key role in deciding what message to convey. For example, if someone wants to retell a story, the aim of retelling will influence the content being produced. People who retell a story with the purpose of being entertaining tend to produce fewer story events but more emotion words compared with those individuals who have been instructed to be accurate (Dudukovic et al., 2004).

The effect of situational factors on communication extends to the paralinguistic and nonverbal aspects of communication, such as speech rate and gesturing. For instance, people who are engaged in a conversation will adjust their speech rate to match that of their interlocutor (Freud et al., 2018; Schultz et al., 2016). Other studies have demonstrated that the visibility and shared knowledge between interlocutors affect the frequencies of gestures and what types of gestures are produced (Alibali et al., 2001; Cochet & Vauclair, 2014; Hilliard & Cook, 2016).

The literature reviewed above provides converging evidence that communication behaviors are flexible and sensitive to the current speaking contexts. However, what remains unclear is whether the speaking situation affects communication in terms of basic communication methods. Specifically, do people’s relative preferences for two communication methods—depiction and description—shift along with changes in communication context? Although depiction, together with description and indication, is considered to be a basic communicating method, it has received scant attention from most language theories (Clark, 2016). In the current study, we fill this omission by comparing the frequency of the use of depiction and description as a function of communication context. The goal is to improve our understanding of different communication methods and thereby contribute to incorporating depiction into current language theories.

There is a clear contrast between depiction and description. Imagine you are talking with your friend about how a male character in a television show knocks on his neighbor’s door. You can use depiction by imitating the character’s behavior as follows: *You lift your hand up and knock on an imagery door three times while saying “knock, knock, knock, Penny,” and then you repeat the whole procedures two more times.* Conversely, you can also use description and say, “*His knock consists of three knocks before stating his neighbor’s name, and then he repeats this process two additional times.*” As we can see from the example, depiction is *showing* other people what an event looks like, sounds like, or feels like, whereas description is *telling* others about an event using the knowledge of a language or a code (Clark, 2016).

Depiction consists of several subtypes, including iconic gestures, facial gestures, direct speech, full-scale demonstrations, and make-believe plays (Clark, 2016). In the current research, we examined the effects of situational factors on the use of direct speech (e.g., *Mary said: “I am hungry*.”) and indirect speech (e.g., *Mary said that she was hungry*.”). Reported speech is of interest to the current study for two reasons. First, direct speech and indirect speech belong to contrasting communication methods. Direct speech is a type of depiction, whereas indirect speech is description (Clark, 2016). Second, direct and indirect speech convey complete messages and can be used interchangeably. Other kinds of depictions, such as iconic gestures, are seldom used independently. The information from the nonverbal channel (e.g., gestures) must be integrated with the verbal channel in order to fully understand a speaker’s intention. For example, one can say, “*I caught a fish of this size!*” while indicating the size of the fish with hands. Previous studies therefore have focused more on how depiction helps description to achieve the communicator’s intentions (de Ruiter et al., 2012; Kita, 2000). Less is known about how people use different methods in specific circumstances to achieve communicative goals. Investigating this question helps to improve our understanding of different communication methods and to reveal how they vary across communicative functions. Understanding these differences also contributes to confirming the necessity of treating depiction as a communication method and therefore as a topic of research in its own right.

To our knowledge, only two studies thus far have investigated how situational factors affect the use of direct speech and indirect speech. In one seminal study, participants watched movie segments and then retold the stories for different purposes. The results show that people use direct speech more frequently when they were asked to be amusing than when they were told to tell accurate stories (Wade & Clark, 1993). A more recent study further demonstrates that people use more direct speech in a dialogue context than in a monologue context (Bavelas et al., 2014). These two studies support the idea that the use of direct speech is sensitive to the aims of communication (e.g., to be amusing or to be accurate) and is also dependent upon the absence/presence of an interlocutor. Inspired by Bavelas et al.’s (2014) work, we investigate here whether the characteristics of interlocutors/recipients play a role in the choice between direct and indirect speech in a narrative context. Given the pervasive nature of psychological distance, a key dimension on which communication situations differ is the psychological distance that the interlocutors appear to be from each other. More insight into the relationship between psychological distance and reported speech will lead to a more detailed description of the flexibility of communication in terms of the methods that are used.

Prior research suggests that people communicate differently with psychologically proximal others as opposed to psychologically distant others. The first line of evidence to support this claim comes from media studies. There are findings showing that people prefer a symbolic (e.g., words) over analogical (e.g., pictures) medium when psychological distance increases. For example, in a series of experiments investigating the use of verbal and pictorial messages, participants were asked to send a message either to psychologically distal others or to proximal others. It shows that psychological proximity leads to an increasing preference of verbal messages relative to pictorial messages (Amit et al., 2013).

Psychological distance has also been observed to influence language use by increasing the level of abstractness. In a recent study, researchers manipulated psychological distance by varying audience size. An increase in audience size is associated with a correspondingly larger social and spatial distance. Participants were motivated to write a self-description either for 50 people or for one person. When the size of the audience increased, the level of abstractness of the descriptions increased correspondingly (Joshi & Wakslak, 2014). Similar results have been obtained when researchers directly manipulate spatial distance and social distance (Joshi et al., 2016; Stephan et al., 2010). Bhatia and Walasek (2016) extended this effect to temporal dimension and beyond the laboratory setting. After analyzing two large Twitter and newspaper databases, they found that people use more concrete language when they refer to near events than when speaking of distant events.

Just as from verbal communication, nonverbal communication is influenced by psychological distance. One study about iconic gesture use is of particular relevance to the current research. Like direct speech, iconic gestures belong to the category of depiction. Iconic gestures are considered as an embodied form of verbal statement (Hostetter & Alibali, 2008). The use of iconic gesture is affected by temporal distance between interlocutors. In Wessler and Hansen’s (2017) study, participants were assigned to the role of a job interviewer. This job would either begin 1 week later (temporally proximal) or 1 year later (temporally distal). The participants were subsequently instructed to interact with an applicant. It was found that participants displayed more imitation of the iconic gestures in the temporally close condition than in the temporally distant condition (Wessler & Hansen, 2017). This result is in line with previous observations that people prefer to use more pictorial and embodied forms of communication when they feel temporally close to the interlocutors.

Taken together, these findings inform us that psychological proximity encourages the use of communication modes that are relatively more analogical, concrete and rich in nonverbal information. What is the mechanism underlying this effect? The rationale behind these observations is that people traverse distance by using either low level or high level mental construals (Trope & Liberman, 2010). Construal level theory (CLT) assumes that a high level of construal contains relatively abstract, decontextualized and essential aspects of events or objects. A low level of construal, instead, captures concrete, contextualized and secondary aspects. High construal level features are more stable and less prone to change across distance. Therefore, distant communication focuses more on conveying the relatively abstract and essential information about a situation, whereas proximal communication provides more context dependent and concrete information.

In the present study, we predicted that psychological distance would result in a contradictory impact regarding the relative use of direct and indirect speech. Psychological proximity should lead people to use direct speech more compared with psychological distance. On the other hand, psychological distance should lead people to use indirect speech more often. There are two reasons for this prediction.

First, using direct speech requires speakers to take a first-person perspective, whereas using indirect speech requires a third-person perspective. According to CLT, the first-person perspective induces less distance than the third-person perspective and thus elicits a lower level of construal on the part of the speaker. Meanwhile, communicating with recipients who are psychologically near will also activate a lower level of construal (Trope & Liberman, 2010). Therefore, we predicted that speakers will used direct speech more often when recipients are psychologically proximal than distal.

The second reason for the prediction is that direct speech is thought to convey more nonverbal information than indirect speech. By depicting the original speaker’s nonverbal information, direct speech creates a physical scene analogous to the original scene (Clark, 2016). Indirect speech, on the other hand, provides a description of the original scene in a symbolic way. Previous studies have shown that people prefer an analogical way (e.g., pictures) over a symbolic way (e.g., words) when an interaction partner is psychologically proximal. Drawing on this logic, it is reasonable to assume that people would use direct speech more frequently when communicating with psychologically proximal others. In the current research, we manipulated three dimensions of psychological distance—social distance (Experiment 1), temporal distance (Experiment 2), and spatial distance (Experiment 3)—to examine our hypotheses. We predicted that people would show an increase in the use of direct speech when they communicate with psychologically proximal others compared with psychologically distal others.

## Experiment 1

The goal of Experiment 1 was to investigate the effect of social distance on the use of direct and indirect speech. We predicted that participants who were assigned to the socially proximal condition would produce a higher rate of direct speech than participants who were assigned to the socially distal condition.

### Method

#### Materials

A 5-minute movie named “One-Minute Time Machine” was used. The movie included a male protagonist and a female protagonist. The male protagonist had a time machine. The story was about how he used the time machine to travel one minute back in time every time he said something wrong while trying to make a good impression on this female protagonist.

#### Participants

Participants were recruited from Erasmus University Rotterdam in the Netherlands. They were reimbursed with course credit of 0.5 hour. G*Power 3 was used to calculate the sample size. We estimated the sample size based on a one-tailed independent *t* test assuming a medium effect size between 0.4 ~ 0.5, alpha = 0.05, and power between 80% ~ 90%. The power analysis showed that a sample size between 102 and 216 was needed to detect the effect. We therefore decided to collect a maximum 220 participants (110 participants per condition). We used a sequential data collection procedure. Sequential analyses provide an efficient way to conduct high-powered studies. By performing interim analyses, researchers can terminate data collection earlier when there is convincing evidence to conclude that an effect is present or absent without increasing the rates of Type 1 error (Lakens, 2014). In the current study, alpha boundaries for interim analyses were calculated using the GroupSeq package with the alpha spending function in R (Lakens, 2014). Given that the alpha spending function does not require the interim analyses to be evenly spaced, we decided to perform analyses at *n* = 100, *n* = 160, and *n* = 220. The adjusted alpha levels for the first, second and third test were 0.023, 0.022, and 0.026, respectively. The smallest effect size of interest for this study is Cohen’s *d* = 0.30. The first interim analysis was planned after collecting 100 valid participants. If the *p* value was smaller than 0.023 or the effect size was below 0.30, data collection would be terminated. Otherwise, an additional 60 valid participants were to be collected. If the *p* value of the second interim analysis was lower than the alpha level of 0.022 or the effect size was smaller than 0.30, date collection would be terminated. Otherwise, a final 60 valid participants were to be recruited. In Experiment 1, data collection was terminated after the first interim analysis because the *p* value was smaller than 0.023. Therefore, the final sample size in Experiment 1 was 100 participants (71 females, average age = 19.89, range = 18–28). This study was approved by the Ethics Committee of Psychology at the Erasmus University Rotterdam.

#### Procedure

Data collection was carried out partially in a sound-attenuated room in the Erasmus Behavioral Lab and partially online via Qualtrics. Consent with agreement to video recording were obtained before participants started the experiment. Participants were informed that their task was to watch a movie clip and then retell the story. They needed to pay close attention to what happened in the movie in order to retell it in detail afterwards. After participants declared that they had fully understood the instructions, the experimental movie clip was shown on the computer screen. Following this, participants were presented with instructions specifying how they should retell the story. They were first instructed to imagine a communication scenario where they told a story to an imagined addressee. To manipulate social distance, half of the participants were instructed to imagine retelling the story to a good friend. The other half were instructed to imagine retelling the story to someone they met for the first time (i.e., a stranger). Upon completion of the retelling task, participants answered a manipulation check question. They reported whether they complied with the instructions and telling the story to a good friend or a stranger.

#### Data preparation and coding

Invalid recordings were discarded prior to data analyses. Recordings were considered invalid if they fell into one of four categories: (1) when participants reported that they had not followed the instructions to retell the story to a friend or a stranger were excluded (*n* = 9), (2) when participants withdrew from the experiment before finishing all tasks (*n* = 26), (3) when recordings were difficult to transcribe due to noise (*n* = 1) or recording device malfunction (*n* = 13), and (4) when recordings did not contain any reported speech (*n* = 17). This resulted in the removal of data from 66 participants (45 participants from the socially proximal condition).

All retellings were transcribed verbatim and then segmented into utterances. Utterance was defined as a main clause together with dependent clauses (Bishop & Donlan, 2005). Main clauses linked by coordinating conjunctions such as “and,” “so,” “but” were coded as separate utterances unless the subject of the clause was omitted. Utterances that omitted obligatory elements of a clause structure were treated as a separate utterance (Bishop & Donlan, 2005). Reported speech that contained more than one main clause were treated as several individual utterances if the main clause itself met the criteria for a new utterance (Frizelle et al., 2018). For example, “She said ‘My 5-year-old niece likes science. This is not science.’” would be treated as two utterances. Incomplete utterances, self-corrections, and repetitions of a previous utterances, utterances that were not related to the content of the video (e.g., I don’t remember what he said exactly) were discarded.

Next, all quotations were categorized as direct or indirect speech based on deictic terms indicating perspective (e.g., pronouns, verbs, space reference, and time reference). Quotations that were from the character’ point of view were coded as direct speech, whereas quotations from the observer’s point of view were coded as indirect speech. For quotations that could not be classified by the above-mentioned criteria, the coders would listen to the recording for speaker’s intonation (Wade & Clark, 1993). If there was any change in the speaker’s voice compared with her or his normal voice, the utterance was coded as direct speech. Otherwise, it was treated as indirect speech (Wade & Clark, 1993). Two coders who are not involved in data collection and are blind to the manipulation were recruited to code all recordings. The interrater reliability between two coders was 0.82, which indicated very high agreement (Landis & Koch, 1977). Disagreements between two coders were discussed and resolved before data analyses.

### Results and discussion

The dependent variable was the number of utterances of direct speech as a proportion of the number of utterances of reported speech that a participant produced. Data analyses were performed using a mixed-effects model with the lme4 package in R (Baayen et al., 2008). Social distance was treated as the fixed effect and participants were treat as random effects. The proportion of direct speech in the socially proximal condition (*M* = 55.38%, *SD* = 0.31) was higher than in the socially distal condition (*M* = 35.51%, *SD* = 0.41). Model comparison revealed a significant effect of social distance on the use of direct speech, *χ*^*2*^(1) *=* 7.34, *p =* 0.007, Cohen’s *d* = 0.55. The results showed that social proximity led to an increase in the proportion of direct speech among reported speech utterances. When reporting previous utterances, participants were more likely to use direct speech when they told the story to a friend than to a stranger, which supports our hypothesis Tables 1, 2, and 3.

**Table 1:** Descriptive information (*M* and *SD*) of narrations from the socially proximal and distal condition

Condition	Number of utterances	Number of words	Number of utterances of direct speech	Number of utterances of indirect speech
Proximal	44.42 (28.25)	488.18 (268.86)	10.20 (15.21)	3.40 (3.65)
Distal	34.12 (24.05)	395.50 (250.45)	5.82 (9.60)	3.00 (2.71)

**Table 2:** Descriptive information (*M* and *SD*) of narrations from the temporally proximal and distal conditions

Condition	Number of utterances	Number of words	Number of utterances of direct speech	Number of utterances of indirect speech
Proximal	39.00 (29.78)	400.53 (263.58)	9.39 (15.00)	2.20 (2.79)
Distal	36.84 (28.34)	380.74 (243.41)	7.44 (12.04)	3.88 (6.80)

**Table 3:** Descriptive information (*M* and *SD*) of narrations from the spatially proximal and distal conditions

Condition	Number of utterances	Number of words	Number of utterances of direct speech	Number of utterances of indirect speech
Proximal	47.66 (28.45)	510.64 (275.46)	10.6 (13.89)	4.18 (3.86)
Distal	40.64 (29.75)	437.12 (253.04)	10.8 (17.34)	3.68 (7.88)

## Experiment 2

The results of Experiment 1 indicated that people evaluated a recipient’s social distance and constructed their narrations correspondingly. This result supports the prediction of CLT that people in social proximity favor an analogical way (direct speech) rather than a symbolic way (indirect speech) of communication. In Experiment 2, we focused on another dimension of psychological distance, namely temporal distance. We predicted that participants in a temporally proximal condition should produce a higher rate of direct speech than participants in a temporally distal condition.

### Method

#### Materials and participants

The same video “One-Minute Time Machine” as in Experiment 1was used. A power analysis showed that a maximum 220 participants were needed. A sequential analysis was carried out along data collection. The alpha level for each interim analysis was as the same as those in Experiment 1. The first interim analysis showed that there was no significant difference in the use of direct speech between temporally proximal and distal condition, χ^2^(1) *=* 2.34*, p =* 0.13. The *p* value was larger than the alpha level of 0.023. The effect size was Cohen’s *d* = 0.30. Therefore, another 60 participants were collected. The second analysis showed a significant difference in the use of direct speech between two conditions, χ^2^(1) *=* 4.67, *p =* .031. The *p* value was larger than 0.022. Therefore, the final 60 participants were collected. The final sample size consisted of 220 participants (184 females, two others, average age = 19.81, range = 17–29).

#### Procedure

Data collection was performed partially in the Erasmus Behavioral Lab and partially online via Qualtrics. The procedures of Experiment 2 were identical to those of Experiment 1, except for two adjustments. First, a different pair of instructions was shown to participants. In Experiment 2, after viewing the movie, participants were instructed to retell what happened in the video either to a temporally distant other or a temporally proximal other. Second, the manipulation check question was changed correspondingly. Participants were asked to report when the other participant would watch his or her video. Specific instructions in temporally distal and proximal conditions were as follows:

The temporally distal condition:

*This project investigates “Information transfer between individuals.” It consists of two parts. You are now participating in the first part. Please retell what went on in the movie as detailed as possible. In the second part, another participant will watch your video* *6 months later. After watching, he/she will retell the story again. Please keep in mind when the other participant will watch your video while you are retelling the story.*

The temporally proximal condition:

*This project investigates “Information transfer between individuals.” It consists of two parts. You are now participating in the first part. Please retell what went on in the movie as detailed as possible. In the second part, another participant will watch your video* *tomorrow. After watching, he/she will retell the story again. Please keep in mind when the other participant will watch your video while you are retelling the story.*

#### Data preparation and coding

The same exclusion criteria were applied in Experiment 2 as in Experiment 1. Recordings were excluded from the analyses when (1) when participants failed to answer the manipulation check question correctly (*n* = 9); (2) when participants withdrew from the experiment before finishing all tasks (*n* = 59); (3) when recordings failed due to device malfunction (*n* = 17); and (4) when there was no reported speech in the recordings (*n* = 35). A total of 120 participants was removed from data analyses. The remained recordings were then transcribed and coded following the exact same protocol used in Experiment 1. The same two coders from Experiment 1 coded all recordings independently. The inter-rater reliability between two coders were 0.86, which indicated very high agreement (Landis & Koch, 1977). Disagreements were discussed and resolved before data analyses.

### Results and discussion

We ran a mixed-effects regression model with temporal distance as a fixed factor and participants as random factors. Participants used more direct speech in the temporally proximal condition (*M* = 54.04%, *SD* = 0.42) than in the temporally distal condition (*M* = 41.88%, *SD* = 0.37). Model comparison revealed a significant effect of temporal distance on the rates of direct speech, χ^2^(1) *=*5.10*, p =* 0.024, Cohen’s *d* = 0.31. This result supports our prediction that people in temporal proximity produce a higher rate of direct speech compared with people in temporal distance, whereas people in the temporal distance condition produced a higher rate of indirect speech compared with those in the temporal proximity condition. It is in line with previous studies of gesturing and the abstractness of language and supports the view that feeling temporally close to a recipient would lead to a preference for the analogical way of communicating.

## Experiment 3

The results of Experiment 1 and Experiment 2 showed that social distance and temporal distance influence the preferences of communicating methods in the same manner. A feeling of social and temporal proximity was associated with a more frequent use of direct speech. The goal of Experiment 3 was to investigate the effect of spatial distance on the use of direct and indirect speech. We predicted that participants who were assigned to the spatially proximal condition would produce a higher rate of direct speech than would participants who were assigned to the spatially distal condition.

### Method

#### Material and participants

The same video “One-Minute Time Machine” was used in Experiment 3 to elicit reported speech. Sample size was determined by power analysis as well as a sequential analysis, similar to Experiment 1. In the first interim analysis, we did not find a significant difference in the use of direct speech between the spatially proximal and distal condition. In addition, the effect size was 0.20. This is smaller than the minimum effect size 0.30. Therefore, data collection was terminated after collecting 100 valid participants (69 females, 1 other, average age = 19.96, range = 17–25).

#### Procedure

Data collection was performed in the Erasmus Behavioral Lab. The procedure of Experiment 3 was similar to those of Experiment 2. After watching the movie, participants read a cover story in which we manipulated spatial distance between them and the anticipated recipients. Upon completion of the storytelling task, participants were asked to recall the location of the other participant who would watch their video. The specific instructions that manipulate spatial distance were as follows:

The spatially proximal condition:

*You are participating in a project investigating “Information transfer between individuals.” Two research groups are collaborating on this project. One group is from Rotterdam, the Netherlands, and the other group is from Nebraska, U.S. Now, please retell what went on in the movie as detailed as possible. Another participant who is in* *Rotterdam* *will watch your video. After watching, he/she will retell it again. Please keep in mind this participant’s location while you are retelling the story.*

The spatially distal condition:

*You are participating in a project investigating “Information transfer between individuals.” Two research groups are collaborating on this project. One group is from Rotterdam, the Netherlands, and the other group is from Nebraska, U.S. Now please retell what went on in the movie as detailed as possible. Another participant who is in* *Nebraska* *will watch your video. After watching, he/she will retell it again. Please keep in mind this participant’s location while you are retelling the story.*

#### Data preparation and coding

Data cleaning was performed in the same manner as with Experiments 1 and 2. Recordings were excluded from analyses when (1) participants did not answer the manipulation check question correctly (*n* = 7); (2) recordings failed due to device malfunction (*n* = 1); and (3) recordings did not contain any reported speech (*n* = 12). This resulted in a total 20 of recordings were removed. The remined recordings were transcribed and coded. All recordings were coded by two independent coders. The interrater reliability was 0.85, which indicated a very high agreement (Landis & Koch, 1977). Disagreement was discussed and resolved before data analyses.

### Results and discussion

We ran the mixed-effects regression in R with spatial distance as a fixed factor and participants as the random factors. Participants did not differ in the extent to which they used direct speech in the spatially proximal (*M* = 49.39%, *SD* = 0.36) and distal condition (*M* = 56.96%, *SD* = 0.38). Model comparisons showed spatial distance did not affect the rates of direct speech in two conditions, χ^2^(1) *=*1.04*, p =* .31, Cohen’s *d* = 0.20. It is a bit puzzling that no significant difference in the use of direct and indirect speech was detected, given that different dimensions of psychological distance has been argued to be related and to have similar effects on various cognitive processes. We return to this unexpected outcome in the general discussion.

## General discussion

The current study aimed to investigate whether psychological distance has an effect on the use of different communication methods: depiction (i.e., direct speech) and description (i.e., indirect speech) in a narrative context. In three experiments, participants watched a short video and then retold what happened in the video to either psychologically proximal others or psychologically distal others. The results of Experiment 1 (social distance) and Experiment 2 (temporal distance) showed that participants were more likely to use direct speech instead of indirect speech when communicating with psychologically proximal others. Unexpectedly, in Experiment 3 (spatial distance), no significant difference in the use of direct speech between spatially proximal and distal condition was detected. We will discuss the theoretical implications of this work and possible explanations for the nonsignificant results in Experiment 3.

The current study is based on CLT and Clark’s (2016) proposal about methods of communication. CLT argues that the lesser the psychological distance is, the more likely a speaker will communicate in an analogical way. Depiction, as stated by Clark (2016), is a physical analogy of the original scene and characterized by rich simulations (Yao et al., 2011, 2012). Taken together, CLT predicts that psychological proximity should encourage the use of depiction. Consistent with this assumption, we found that participants took recipients’ distance into account when constructing a narration. They used depiction (i.e., direct speech) more often when the recipients were psychologically proximal compared with when they were psychologically distant. On the other hand, participants used description (i.e., indirect speech) more frequently when the recipients were psychologically distant than when they were proximal. These results are also in line with existing work showing that psychological distance attenuates the use of pictures (Torrez et al., 2019) and temporal distance attenuates the imitation of iconic gestures from an interaction partner (Wessler & Hansen, 2017).

This study enhances our understanding of direct and indirect speech. Though these two reporting styles occur frequently in daily communication, limited resources have been devoted to investigating them empirically. Existing evidence shows that direct speech, as opposed to indirect speech, increases the vividness and the comprehensibility of stories (Groenewold et al., 2014; Wade, & Clark, 1993). In two experiments, we observed that people’s choice to use direct or indirect speech in a description varied as a function of psychological distance. This indicates that direct speech and indirect speech differ from each other in terms of the communication function they fulfil. Direct speech can be used to reflect the closeness between speakers and recipients, whereas indirect speech is used to reflect the distance.

This study also has implications for the construal level theory due to the nonsignificant result in Experiment 3. In Experiment 3, we examined the relationship between spatial distance and the use of direct and indirect speech. Unexpectedly, we found that participants did not differ in the extent to which they use direct speech when communicating to a recipient who is either spatially near or far. This result does not support the CLT’s prediction that spatial proximity increases the use of low-level construals and the analogical way of communication. Besides that, this result is also in contrast with existing evidence showing that spatial distance reduces the use of pictorial communication (Amit et al., 2013). Presumably, the characteristics of different psychological distance dimensions or the nature of our design can account for this puzzling result.

First, it is possible that the strengths of different psychological dimensions might vary. Spatial distance has no or a weak effect on the use of direct speech and indirect speech. Though it has been argued that different dimensions of distance are interrelated, the possibility that some distance dimensions have greater influence than others has not been ruled out. Which dimension is more fundamental is still a matter of dispute. Spatial distance, for example, could be the more basic dimension because children acquire the concept of spatial distance earlier as it is highly relevant to them being able to move around safely (Boroditsky & Ramscar, 2002). In support of this assumption, researchers found an asymmetrical relationship between spatial distance and other psychological distances. After receiving a distal prime on the spatial dimension, people perceive greater distance on social, temporal, and hypothetical dimensions, but not the other way around (Zhang & Wang, 2009). Indeed, inconsistent results among different psychological distance dimensions have been observed in the field of moral evaluation. For example, in one study people were asked to judge moral transgressions of different social and temporal distances. They judged socially distant transgressions more harshly but not temporally distant ones (Žeželj & Jokić, 2014). Taken together, these studies show us a possibility that different psychological distance dimensions might have various effects. However, more research is needed to determine which dimension is more primary and has the strongest effect. In the current study, we did not observe an effect of spatial distance, whereas Žeželj and Jokić (2014) failed to observe an effect of temporal distance. Therefore, whether we can observe an effect could also be depending on both the strength of a psychological distance dimension and the nature of the cognitive activities. Back to our study, the nonsignificant results in Experiment 3 could be caused by the intrinsic differences that lie in different psychological dimensions. Further research is needed to fully understand how different dimensions interact with each other and whether different dimensions have similar effects on language use.

Second, the nonsignificant result in Experiment 3 could also be due to the nature of our design. In Experiment 3, we instructed participants to retell the story. They were told that another participant who was either spatially near or spatially far away would watch their video. However, the communication between participants and anticipated recipients was in an online context in the form of digital communication (Norman et al., 2016). Various digital communication tools make it possible for us to communicate with spatially distal people simultaneously. This means that perceived spatial distance might be attenuated in online contexts (Norman et al., 2016). Furthermore, the use of digital communication technologies allows people to experience discrepancy between different psychological dimensions more frequently (Norman et al., 2016). Therefore, among the three dimensions, spatial distance is the most prone to be influenced by communicating media (Sungur et al., 2017). The potential influence of digital communication on psychological distance has caught the attention of researchers. A recent study investigating the relationship between hypotheticality and spatial distance in online message processing revealed inconsistent results between experiments. In Sungur et al.’s (2017) Experiment 3, it is observed that participants in the spatially near condition tended to believe that the event described in the online message was more likely to happen. In their Experiment 4, however, participants’ expectation on spatial distance of the online message’s source was not influenced by the probability of the event described in the message. The researchers argued that online communication allowed more inconsistencies between psychological distance dimensions. Therefore, the congruency effect might be less strong in online contexts than in offline contexts (Sungur et al., 2017). In our study, participants and anticipated recipients “communicate” through video-recorded messages. Participants may not perceive the distal location as far as we expected. The perceived distance between two conditions was not great enough to induce a difference in the use of direct and indirect speech.

To sum up, as discussed earlier, inconsistent results from different psychological distance dimensions suggest that the strength of different dimensions may vary, with spatial distance being the weakest dimension. It is also possible that the online communication setting in Experiment 3 reduced perceived spatial distance between speakers and recipients.

This study is not without limitations. First, participants told the story to either an imagined or an anticipated recipient. This is more of a monologue-like setting since the recipient is invisible and there is no interaction between them (Bavelas et al., 2014). Depiction, as argued by Clark (2016), “is to show others what it looks or sounds or feels like” (p.342). This definition emphasizes the importance of a visible and interacting recipient. Indeed, it has been shown that people use more direct speech in a dialogue condition than in a monologue condition (Bavelas et al., 2014). Thus, one needs to be cautious when generalizing these results to other settings such as: a face-to-face dialogue condition, a monologue condition, or a written communication condition. Second, we only tested the influence of psychological distance on use of direct and indirect speech. How the use of direct or indirect speech affects the perceived psychological distance was not examined. Actually, existing literature suggests that certain aspects of language such as the level of politeness (Stephan et al., 2010), the voice (passive and active) of a sentence (Chan & Maglio, 2020), and even the type of vowels (Maglio & Feder, 2017) will influence perceived psychological distance. It would be interesting for future studies to test whether reported speech both reflects and regulates psychological distance. Third, in Experiment 3, participants did not differ in the extent to which they use direct speech in a spatially proximal or a spatially distal condition. Possible explanations were discussed. We could not, however, disentangle these two assumptions in the current study. It will be an interesting topic for researcher to further investigate the interaction between psychological distance communication in different settings. To conclude, this study reveals that different psychological distance dimensions may have various effects on people’s preferences of communication methods.
